# Milestones of the last 10 years: CNS cancer

**DOI:** 10.1007/s12254-016-0309-x

**Published:** 2017-01-25

**Authors:** Christine Marosi, Matthias Preusser

**Affiliations:** Division of Oncology, Department of Medicine I, Medical University Vienna, Waehringer Guertel 18–20, 1090 Vienna, Austria

**Keywords:** Updated WHO classification of central nervous system tumors, CNS4+, Glioblastoma multiforme, Anaplastic glioma, Diffuse glioma, IDH1 mutation, Meningioma

## Abstract

For neuro-oncologists, much was accomplished in the last decade, including the establishment of the first standard of care (SOC) in this field of oncology. New treatment options boosted research in the whole field of neuro-oncology, as well clinical trials, translational and basic research. Accumulated data on molecular–genetic subgroups with distinct clinical outcomes in disease entities led to the establishment of new biomarkers and to the collaborative formulation of a new WHO classification of central nervous system tumors.

For neuro-oncologists, the last decade was a laborious period of time, following the establishment of the first standard of care (SOC) in the field of oncology, e. g., the SOC for the first line treatment of adult patients with newly diagnosed glioblastoma multiforme (GBM) in 2005 [1]. This SOC consisting of maximal feasible safe tumor resection, followed by 6 weeks of radiochemotherapy with temozolomide and adjuvant temozolomide became the treatment paradigm in the world and this resulted in prolonging the survival of GBM patients for the first time after decades of frustrating stagnancy [2, 3]. This boosted research in the whole field of neuro-oncology, as well clinical trials as translational and basic research.

Accumulated data on molecular–genetic subgroups with distinct clinical outcomes in disease entities led to the establishment of new biomarkers and to the collaborative formulation of a new WHO classification of central nervous system tumors *CNS4+* which was published in June 2016 [4–6]. This new classification of CNS tumors integrating classical histological classification, grading, and immunohistochemical and molecular–genetic data is a major milestone in the development of neuro-oncology and will allow a better characterization of tumors as better assignment of treatments, will most probably allow more rapidly informative trial results on better defined, more homogenous patient populations to be obtained. This new classification will undoubtedly speed up scientific progress in all domains of neuro-oncology in the following years.

## Glioblastoma multiforme

The prolongation of survival achieved by adding temozolomide to radiotherapy and performing adjuvant chemotherapy with temozolomide is not equally distributed among all patients with GBM, but restricted to those in whose tumors the promoter of the DNA repair enzyme methylguanine-methyltransferase (MGMT) is methylated, allowing the efficacy of alkylating chemotherapy [7, 8]. This is also true for all other gliomas or other tumors where MGMT promoter methylation allows alkylating chemotherapies to be effective.

In the following years, a number of trials have been conducted in order to improve the actual SOC in GBM, but no further breakthrough with drug therapy has yet been achieved. Neither intensifying the dose, nor prolonging the exposure to temozolomide [9] nor adding the integrin-inhibitor cilengitide [10, 11], nor the anti-VEGF antibody bevacizumab [12, 13] was able to prolong the survival of patients as compared to control patients treated according to the current SOC. Most remarkably, advances in surgery and radiotherapy lead to a prolongation of the median overall survival to 18–20 months in the most recent trials. However, there was a positive study using an electrical device for maintenance of remission possibly leading to a survival gain of a further 3 months; Novocure TTF [14] delivers alternating electrical fields to the brain, but the mode of action of this therapy remains unclear, as well as the burden to quality of life and the cost effectiveness of this device. Currently, several trials are investigating the addition of immune therapy as dendritic cell vaccines and immune checkpoint inhibitors in glioblastoma patients.

For elderly patients with glioblastoma, Lawrence et al. analyzed the outcomes of treatment in the Survival, Epidemiology and End Results Database for patients diagnosed with GBM from 2001–2007 in the US and showed that on a population-based level the survival of the elderly with GBM still stagnated at a median duration of 4 months [2]. During the last decade, a number of trials has quasi systematically cleared how to treat glioblastoma in the elderly; however, none of these trials included a formal geriatric assessment—which would greatly help to allocate treatment to patients. Keime Guilbert et al. demonstrated a significant prolongation of overall survival in patients treated with radiotherapy plus supportive care, versus supportive care alone (29.1 weeks for RT vs 16.9 weeks, hazard ratio [HR] 0.47; *P =* 0.002) [15]. Roa et al. had already shown in 2004 that hypofractionated radiotherapy, e. g., 40 Gy/15 fractions over 3 weeks, achieved similar survival data to the standard radiation course with 60 Gy/30 fractions [16]. Furthermore, the NOA-08 trial and the Nordic Glioma trial both explored radiation therapy versus chemotherapy with temozolomide (TMZ) in two different elderly patients cohorts, both showing that alkylating chemotherapy is beneficial only in patients with GBM with methylated MGMT promoter, whereas radiotherapy also prolongs survival in patients whose tumors are unmethylated [17–19]. Finally, Perry presented at ASCO 2016 a collaborative trial showing that combining hypofractionated radiotherapy (40 Gy/3 weeks)with TMZ, followed by adjuvant TMZ was the most effective treatment also in elderly GBM patients [20].

Based on preclinical data showing expression of immune-related molecules and infiltration by immune cells in brain tumors, as well as promising results from animal studies, clinical trials with immunomodulatory substances including vaccines and immune check-point inhibitors are ongoing and outcomes will be reported in the near future [21].

## Anaplastic gliomas WHO III

As diagnostic criteria for WHO III gliomas have changed substantially in the past years and as the interobserver variation in those diagnoses is well-known to be huge, it is difficult to interpret results from previous trials with recent patients.

For patients with WHO grade III gliomas, two pivotal trials led by the EORTC are currently being conducted, exploring timing and sequence of the currently available treatments:

- CODEL trial for patients with WHO grade III gliomas with codeletion of 1p/19q; currently again recruiting, comparing radiotherapy alone to radiotherapy followed with procarbazine, CCNU, vincristine (PCV) and to radiotherapy + concomitant and adjuvant temozolomide. Results of this trial will be available only in a few years.
- The CATNON trial addressing treatment for anaplastic gliomas without 1p/19q codeletion, a four-arm trial with a 2:2 design, exploring radiotherapy (RT) alone, compared to RT ± concomitant and ± adjuvant temozolomide has finished recruitment of 748 patients. The first planned interim analysis at the end of 2015 after a median follow-up of 27 months showed a HR reduction for overall survival (OS) of 0.645 (95% confidence interval [CI] 0.450–0.926; *p* = 0.0014) using adjuvant temozolomide. Whether also concomitant chemotherapy is beneficial in these patients will be known in 2021.

## Diffuse gliomas WHO II

For patients with diffuse gliomas, the management showed the greatest changes with moving away from the common practice consisting in prolonging observation without intervention as long as possible to active treatment using all modalities. Resecting the gliomas as much as possible without causing a neurological deficit precludes transformation into a higher grade glioma. Furthermore, two studies started more than 10 years ago both showed benefit from radiotherapy as well as from chemotherapy in patients with symptomatic low grade gliomas (LGGs). Buckner et al. reported the results of the RTOG 9802 trial showing a survival benefit with PCV added after radiotherapy, resulting in a significant survival gain of patients treated with subsequent PCV, e. g., 7.8 years vs. 13.3 years, respectively (HR 0.59, *p* < 0.003) [22, 23]. In the EORTC trial 22033, patients with LGG were first registered and 477 patients were randomized to either radiotherapy or dose dense chemotherapy with temozolomide at a later time point, when radiologic tumor progression or progression of symptoms occurred [24]. After a median follow-up of 48 months, patients with IDH1 mutation and 1p/19q codeletion had the longest period of progression-free survival (PFS) of 62 months (95% CI 41–not reached), followed by the patients showing IDH mutation only (48 months, range 41–55 months), whereas patients with IDH wild-type had the shortest PFS of 20 months (range 12–26 months). IDH mutated, non-codel patients experienced a longer period of PFS with radiotherapy than with chemotherapy (55.4 vs 36 months, respectively, *p* = 0.013). Quality of life and cognitive performance were examined prospectively in this trial and showed so far no difference in effect of temozolomide chemotherapy or radiotherapy neither on health-related quality of life nor on global cognitive functioning [25].

## Meningiomas

Although approximately 80% of meningiomas can be cured by surgical resection, about 20% of cases progress or recur due to unfavorable location precluding complete resection or histological or molecular features associated with tumor growth and treatment resistance. Approximately half of meningioma cases carry NF2 aberrations and recent studies have identified other recurring molecular alterations such as AKT mutations, SMO mutations, PI3K mutations, TERT promoter mutations, and gene promoter methylation profiles that show strong correlations with tumor location, tumor histology, and clinical course. These findings will most likely translate into updated classification schemes for meningioma in the near future that will integrate morphological and molecular features, similar to recent advancement in the classification of other brain tumors. Ongoing clinical trials are evaluating not only the role of adjuvant radiotherapy after resection of completely resected newly diagnosed grade II meningiomas (ROAM trial, [26]), but are also evaluating cytotoxic chemotherapy with trabectedin for recurrent grade II/III meningiomas (EORTC-13020 trial, NCT02234050) and targeted therapy according to molecular profile in recurrent meningioma of all grades (NCT02523014). Overall, meningioma research has evolved into a highly active and interesting field of research with rapid advancements in molecular understanding and an active clinical trial portfolio that will hopefully lead to improved treatment modalities within the next few years.

## Brain metastases

Brain metastases are the most common intracranial malignant tumors in adults and affect up to 30% of cancer patients. The incidence of brain metastases seems to be increasing over time, probably due to longer survival of cancer patients with new treatment opportunities and improved diagnostic methods. The main strategies of brain metastasis treatment have long been radiotherapy, surgery, and palliative care. However, in recent years molecular treatments with activity in extracerebral tumor types have been shown to also be useful in some patients with brain metastases. Examples include EGFR and ALK inhibitors in non-small cell lung cancer, BRAF inhibitors in melanoma, HER2-targeting agents in breast cancer, immune check-point inhibitors of CTLA4 or PD1 in melanoma or non-small cell lung cancer and bevacizumab in patients with radionecrosis of corticosteroid-refractory brain edema [27]. These novel treatment options are increasingly being considered in brain metastasis patients and may help to significantly improve the prospects of patients affected by this dreadful manifestation of cancer. Hopefully, more clinical trials will emerge that help to define treatment algorithms for evidence-based decision-making regarding optimal combinations and sequence of treatments for brain metastasis patients.
